# Supplementary material for: A high-resolution mRNA expression time course of embryonic development in zebrafish
Source: eLife. 2017 Nov 16;6:e30860. doi: 10.7554/eLife.30860 (PMC5690287; doi:10.7554/eLife.30860)
Supplement: Supplementary file 6. [file elife-30860-supp6.zip › biolayout-clusters-files/Cluster093-genes.html]

Cluster093


# Cluster093: Genes

| Ensembl ID | Gene Name | Chr | Start | End | Biotype |
| --- | --- | --- | --- | --- | --- |
| ENSDARG00000097323 | ENSDARG00000097323 | 11 | 17888830 | 17896268 | protein\_coding |
| ENSDARG00000098475 | arr3b | 14 | 32497796 | 32509214 | protein\_coding |
| ENSDARG00000010933 | cacna1fa | 8 | 44809458 | 44874531 | protein\_coding |
| ENSDARG00000012297 | cnga3b | 1 | 10087363 | 10092634 | protein\_coding |
| ENSDARG00000087873 | eevs | 6 | 43440538 | 43451477 | protein\_coding |
| ENSDARG00000087059 | fam213ab | 12 | 47908082 | 47912620 | protein\_coding |
| ENSDARG00000030215 | matn1 | 19 | 43718982 | 43742683 | protein\_coding |
| ENSDARG00000031952 | mb | 1 | 54460292 | 54467434 | protein\_coding |
| ENSDARG00000007480 | rpe65a | 2 | 2316199 | 2331029 | protein\_coding |
| ENSDARG00000027236 | rs1a | 11 | 29997172 | 30004914 | protein\_coding |
| ENSDARG00000068474 | si:dkey-94e7.2 | 2 | 30737537 | 30742887 | protein\_coding |
| ENSDARG00000103504 | si:dkey-97i18.6 | 16 | 31682465 | 31698687 | processed\_transcript |
| ENSDARG00000075295 | tulp1a | 23 | 5529820 | 5559815 | protein\_coding |
| ENSDARG00000038770 | zgc:103625 | 3 | 49034327 | 49039922 | protein\_coding |
